# Heat stress enhances VLDL secretion in chicken ovarian follicles to potentiate its impact on follicular cell survival and maturation

**DOI:** 10.1016/j.psj.2025.106137

**Published:** 2025-11-19

**Authors:** Petnamnueng Dettipponpong, Mei-Ying Sin, Yu-Hui Chen, Chuen-Yu Cheng, San-Yuan Huang, Ling-Chu Chang, Shuen-Ei Chen

**Affiliations:** a Department of Animal Science, National Chung Hsing University, Taichung 40227, Taiwan; b Department of Animal Science and Fisheries, Rajamangala University of Technology Lanna, Lampang, Thailand; c Center for Molecular Medicine, China Medical University Hospital, Taichung 406040, Taiwan; d Research Center for Cancer Biology, China Medical University, Taichung 406040, Taiwan; e Cancer Biology and Precision Therapeutics Center, China Medical University, Taichung 406040, Taiwan; f Department of Animal Science and Biotechnology, Tunghai University, Taichung 407224, Taiwan; g The iEGG and Animal Biotechnology Center and Rong Hsing Research Center for Translational Medicine, National Chung Hsing University, Taichung 40227, Taiwan; h i-Center for Advanced Science and Technology (iCAST), National Chung Hsing University, Taichung 40227, Taiwan

**Keywords:** Heat stress, Chickens, Follicle maturation, VLDL secretion, Cell death

## Abstract

Chicken ovaries possess the machinery of VLDL production in response to heat stress (HS). The study sought to define the role of VLDL secretion by hierarchical follicles along their development under HS. HS (42°C) for 8 hr (HS8H) or HS3H and following recovery at 37°C to 16 hr (3H13R) upregulated PCNA and/or IL-1β expressions in granulosa (GC) and theca (TH) cells and augmented progesterone (P4) and estradiol (E2) production, respectively. Inhibition of VLDL production by Lomitapide and Mipomersen suppressed P4 secretion at 3H13R and HS8H, but differentially affected E2 production, leading to altered secretion fashions under thermoneutral conditions (37°C, NC) and during post-HS recovery, suggesting interfered steroidogenic competence toward maturation. HS regardless of duration and recovery or interference with VLDL secretion under NC provoked ROS and MDA accumulation in both cell types. GCs transiently increased VLDL secretion at HS3H and showed overt cell death at 3H13R, while TH cells sustained VLDL production and retained viability. Prolonged HS over 8 hr impaired viability of both cell types with disparate responses in lipid dynamics; constantly depressed neutral lipids and cholesterol in GCs, while TH cells showed increased neutral lipids before HS8H but rapid dissipation to HS16H and suppressed cholesterol at HS8H. Surprisingly, Lomitapide and Mipomersen rescued TH cell viability at HS8H and 8H8R in association with alleviated lipid, MDA, and ROS accumulations, whereas GCs exhibited improved viability at HS8H, but not at 8H8R and 3H13R in couple with worse depletion of neutral lipids and cholesterol, suggesting that HS operates at VLDL production to alter cellular lipid dynamics to potentiate cell death, while TH cells are more thermoresistant due to a proficient adaption in lipid disposal. In conclusion, HS enhances VLDL production in hierarchical follicles to augment its impacts on follicular cell fate, but routine secretion of VLDL is obligatory to sustain follicle maturation under normal conditions, in which TH cells are highly agile in lipid remodeling and redox regulation in adaption to heat insults.

## Introduction

Heat stress (HS) is a major environmental factor limiting reproductive performance of laying hens. Exposure to high ambient temperatures, particularly above 35 °C, alters physiological homeostasis leading to oxidative stress, impaired endocrine regulation, acid–base imbalance, immunosuppression, metabolic inflexibility and even behavioral changes particularly feed and water consumption (Rozenboim et al., 2007; Lara and Rostagano, 2013; Kumar et al., 2021). The dysregulations directly or indirectly impede ovarian functions leading to poor egg production and egg quality (Rozenboim et al., 2007; Kim et al., 2024). Most of the ovarian dysfunctions are characterized by impaired follicle maturation as indicated by interfered cell proliferation and steroidogenesis, follicle atresia as indexed by cell apoptosis, and retarded follicle growth leading to limited follicle pools of various classes (Li et al., 2020; Kumar et al., 2021; Yan et al., 2022). Heat stress also enhances hepatic lipid synthesis but limits apolipoprotein B (apoB) secretion, thereby impairing VLDL availability for yolk deposition, and subsequently follicle growth and yolk size (Chen et al., 2006; Lu et al., 2019; Kumar et al., 2021).

In mammals, triacylglycerol-rich lipoproteins are primarily produced in the liver and intestine, where MTTP (microsomal triglyceride transfer protein) and apoB coordinate lipid packaging into lipoproteins for export. However, studies have shown that tissues other than liver and intestine such as the heart, kidney, and placenta also express functional MTTP and apoB for VLDL production to support local lipid homeostasis against triacylglycerol (TAG) accumulation, oxidative damage, and lipotoxicity (Borén et al., 1998; Nielsen et al., 1998; Krzystanek et al., 2010). In avian species, the liver and small intestine are well characterized for apoB-containing lipoprotein production (Griffin et al., 1982; Kirchgessner et al., 1987; Ivessa et al., 2013), while MTTP and apoB expression have also been detected in the yolk sac epithelium of developing chick embryos, where they are associated with yolk lipid transfer to the embryo (Kanai et al., 1996; Eresheim et al., 2014).

Previous transcriptomic studies in cattle confirmed that ovarian follicles possess necessary metabolic pathways to process fatty acids from lipoprotein turnover, uptake, and lipid dissipation, de novo synthesis, and anabolism for storages (Bertevello et al., 2018). These functions support oocyte energy demands, membrane formation, and lipid-based signaling crucial for follicular development and successful reproduction. In mammals, elevated non-esterified fatty acid (NEFA) concentrations in the follicular environment were linked to mitochondrial dysfunction and oxidative stress, leading to impaired oocyte development (Leroy et al., 2005; Aardema et al., 2011). In human luteinized granulosa cells (GCs), functional MTTP and apoB for VLDL production have been reported to secure follicle development and oocyte quality (Gautier et al., 2010). Our earlier works demonstrated that chicken ovarian tissues express functional components of the VLDL assembly machinery. While present in both cell types, apoB was primarily localized in follicular theca (TH) layers and MTTP dominated in GCs (Chen et al., 2025). A series of assessments further confirmed that chicken ovarian tissues assemble and secret generic VLDL. However, the physiological significance of the machinery of VLDL production in the ovary requires further investigations.

In birds, ovarian steroidogenesis is operated through a three-cell model; testosterone, estradiol (E2), and progesterone (P4) being mainly produced in the TH interna, TH externa, and GC layer in the follicle, respectively (Porter et al., 1989). Within the hierarchy, testosterone production increases from the smallest follicle, sustained in the middle classes and finally decreased in the largest follicle, namely F5 < F4 = F3 = F2 > F1, whereas E2 production declines along follicle advancement to ovulation, namely, F5 > F4 > F3 > F2 > F1, and P4 is produced as F5 < F4 < F3 < F2 < F1 (Bahr et al., 1983; Robinson and Etches, 1986; Yu et al., 1992; Johnson, 2000). Consequently, P4 production by GCs is commonly used as a hallmark to assess cell differentiation and follicle maturation, while the role of TH cells tends to be overlooked.

Previously, we demonstrated that the VLDL secretion was reduced by both the MTTP activity inhibitor Lomitapide (Lom) and the apoB antisense oligonucleotide Mipomersen (Mip) in chicken follicular GCs and TH cells, but was increased by thermal stress and oleate stimulation (Chen et al., 2025). Since circulating VLDL is massively targeted to the ovary for yolk deposition (Bacon et al., 1978), follicular tissues tend to be satiated with available lipids and thus are at a high risk for lipid overload. Hence, exporting cargo lipid via VLDL secretion by follicles per se may function in cellular lipid dynamics along their development toward maturation. The present study aims to elucidate the role of VLDL secretion by GCs and TH cells in respect to cell viability and differentiation of follicle development under normal or HS conditions. Intervention of VLDL secretion was carried out by pharmacological inhibition with Lom and Mip, 2 clinical drugs for lipid-lowering medications.

## Materials and methods

### Animal management

All chicken husbandry and experimental procedures were reviewed and approved by The Institutional Animal Care and Use Committee (IACUC) of National Chung Hsing University, Taiwan (IACUC Permit No. 109-134). A flock of Leghorn hens during age 35 to 55 weeks were maintained for the study. Birds were caged individually and fed with a regular soy-and-corn-based layer diet and allowed free access to feed and water throughout the experiment. Feed was placed at 08:30 a.m. in conjunction with a 14L:10D (lights on at 05:00 a.m.) photoschedule.

### Isolation of follicle granulosa and theca cells

To ensure similar ovarian functions, only hens with successful egg production for at least 3 days were used for sampling. In each round of sampling, 1 or 2 hens from the flock during age 35 to 55 weeks were used for follicle collection. Follicle cell preparations from differently aged hens exhibited a similar response in viability under thermoneutral or HS conditions. Since the time of oviposition was not recorded precisely, only the F2 to F5 follicles were used for cell isolation; the F1 follicles were excluded due to possible variations in hormonal status. Granulosa layers were mechanically separated from the ovarian follicles following the method of Gilbert et al. (1977). The sheets of TH layer remaining in follicles were then peeled off and carefully collected with sharp forceps. Collected GC and TH layers were washed twice with PBS, and then digested in M199-HEPES medium containing 200 U/mL type-2 collagenase and 0.3 mg/mL trypsin inhibitor (Sigma-Aldrich, St. Louis, MO, USA) at 37°C for 30 min. The lysates were pooled and then centrifuged at 50 × *g* for 5 min, and cell pellets were resuspended in M199-HEPES medium containing 10 % FBS and seeded in microplates.

### Cell cultures and heat stress treatment

Culture microplates were coated with gelatin (Sigma-Aldrich) at 150 μg/cm^2^ and dried under ultraviolet light for 40 min. Cell suspensions were plated in microplates containing M199 medium 10 % FBS, 100 U/mL penicillin, and 100 μg/mL streptomycin sulfate, pH 7.4, and incubated at 37°C under 5 % CO_2_/95 % air overnight. After replacing with new medium, cells were grown to reach 85 % confluence for HS studies. Heat stress was applied by exposing cells at 42°C for various durations and allowed recovery at 37°C to reach 16 hr as indicated. Cells maintained at 37°C served as a thermoneutral control (NC).

For studies with pharmacological inhibition of VLDL secretion, cells were incubated with 2.5 μM MTTP inhibitor (Lomitapide, Sigma-Aldrich, St. Louis, MO, USA) for 2 hr, followed by replacement with 2.5 μM antisense oligonucleotide for apoB synthesis inhibitor, (Mipomersen, MedChemExpress, Monmouth Junction, NJ, USA) in DMSO (dimethyl sulfoxide; Sigma-Alrich, St. Louis, MO, USA) for another 2 hr. The optimal dosages and specificity of Lom and Mip have been previously investigated (Chen et al., 2025). After washout, cells were subjected to HS treatment and cells and medium were collected at indicated time points for studies. Cell viability was assessed using the CCK-8 kit (ab228554, Abcam, Cambridge, UK).

### VLDL isolation

Harvested culture medium were used for VLDL isolation using NaCl density solution (density = 1.006 g/mL) comprising gentamicin 50 μg/mL, reduced glutathione 0.5 mg/ml, butylated hydroxytoluene (BHT) 50 μg/mL, and phenylmethylsulfonylfluoride (PMSF) 0.35 mg/mL for ultracentrifugation at 148,600 × *g* for 18 h at 14°C (Walzem et al., 1994). The isolated VLDL was further concentrated by ultrafiltration spin-columns with membrane sieve at 5 kDa MW (Millipore, Burlington, MA, USA) and stored under −20°C until use.

### Antibody production

Purified chicken plasma apoB (Nimpf et al., 1988) and a synthetic peptide epitope (CRKVFSTASDSSGSWF corresponding to the carboxy-terminal of the M subunit of chicken MTTP, Thermo Fisher Scientific, Waltham, MA, USA) (Ivessa et al., 2013) were used for antibody production in rabbits and mice, respectively. The immunization procedure, antibody purification, and specificity validation were described previously (Chen et al., 2025)

### Metabolic labeling

The methionine analog, azidohomoalanine (Click-iT^TM^ AHA, Thermo Fisher Scientific, Waltham, MA, USA) was used to trace de novo apoB synthesis and secretion as described previously (Chen et al., 2025). Cells were incubated with AHA (50 μM) overnight and then subjected to HS treatment. Culture medium at indicated time points was collected for VLDL isolation. Isolated VLDL were used for total protein extraction. The newly synthesized proteins with AHA incorporations in VLDL protein extracts were labeled with biotin by the Click-iT® Protein Reaction Buffer kit and Biotin Azide (Thermo Fisher Scientific, Waltham, MA, USA). The standard SDS-PAGE and Western blot procedure were performed using streptavidin-HRP conjugate probing and enhanced chemiluminescence photography for visualization.

### Determination of VLDL-apoB, E2, P4 concentrations, and cellular MDA and cholesterol contents

VLDL-apoB concentrations were measured using a chicken-specific apoB ELISA kit (No. CSB-EL001918CH, CUSABIO, Houston, TX, USA). Progesterone and E2 levels were determined by commercial ELISA kits (No. 0651423, Cayman Chemical, Ann Arbor, MI, USA; No. U0390I036, Fine Biotech, Wuhan, Hubei, China, respectively). Cellular malondialdehyde (MDA) contents were assessed by test kits (Cat. E-BC-K025-M and E-BC-K117-M, Elabscience, Houston, TX, USA) according to the manufacture’s protocols. For cellular total cholesterol determination, total lipids were extracted in chloroform/methanol (2:1; v/v). After drying by nitrogen gas stream, lipids were dissolved in tert‑butyl and Triton X-100/methyl alcohol mixture (1:1 in volume) (Wang et al., 2001) and clinical cholesterol kits (LOT. 21012, Gesellschaft für Biochemica und Diagnostica mbH, Wiesbaden, German) were used for total cholesterol determination.

### MTTP activity analysis

Cell lysates were prepared by homogenization in buffer (100 mM Tris-HCl, 1.5 M NaCl, 10 mM EDTA, 0.5 mM PMSF, 20 μg/mL leupeptin) with 6 rounds of sonication (550 W) on ice for 5 seconds. MTTP activity was then analyzed according the instructions enclosed in the commercial kits (Cat. No. MAK110, Sigma-Aldrich) as reported previously (Chen et al., 2025). The fluorescence was read under the fluorescence microplate reader at Ex/EM: 465/535 (TECAN infinite® 200Pro, AG, Switzerland) for 3 hr. MTTP activity were expressed as a percentage of lipid transfer and calculated with the equation: the percentage of lipid transfer = (arbitrary fluorescence units in assay wells – blank values)/ (total fluorescence units – blank values) x 100.

### Western blot analysis

Cells and isolated VLDL were used for protein extraction with RIPA buffer containing a protease and phosphatase inhibitor cocktail (Cat. No. 78425, Thermo Fisher Scientific, Waltham, MA USA). A mini-gel system (Bio-Rad, Hercules, CA, USA) and SDS-PAGE were used for protein separation and followed by the Western blot analysis. After transfer, the membranes were blocked and probed with an antibody raised against chicken apoB or MTTP-M, or a commercial antibody against PDI (protein disulfide isomerase, sc-74551, Santa Cruz, Dallas, TX, USA), PCNA (proliferating cell nuclear antigen, sc-56, Santa Cruz), StAR (Steroidogenic acute regulatory protein, bs-3570R, Bioss, MA, USA), or IL-1β (interleukin-1β, Cat. AHP941Z, Bio-Rad, Hercules, CA, USA). A horseradish peroxidase (HRP)–conjugated secondary antibody against rabbit or mouse IgG (Cell Signaling Technology, Danvers, MA, USA) was used to interact with the primary antibodies. The signal was captured with the chemiluminescence system by the Clarity™ Western ECL Substrate and quantified using the ChemiDoc^TM^ Touch Imaging System and Image Lab Software (Bio-Rad, Hercules, CA, USA).

### Neutral lipid staining and ROS production

Cells were fixed with 4 % paraformaldehyde for 30 min and stained with BODIPY-^TM^493/503 (1 mg/mL in PBS, Cat. D3922, Molecular Probes, Carlsbad, Calif, USA) for 15 min as described previously (Chen et al., 2020). For reactive oxygen species (ROS) production, MitoSOX^TM^ Red (Lot. 2690484, Thermo Fisher Scientific, Waltham, MA USA) and 2,7-Dichlorodihydrofluorescein diacetate (DCFH; No. 85155, Cayman Chemical, Ann Arbor, MI, USA) were used to stain mitochondrial and cytosolic ROS, respectively. Each reagent was prepared in Hank’s Balanced Salt Solution (HBSS) containing calcium and magnesium. Cells at indicated time points after HS were incubated with MitoSOX^TM^ Red or DCFH (2,7-Dichlorodihydrofluorescein diacetate) in dark at 37°C for 30 min. After wash in PBS for 3 times, the florescence signals were visualized using a florescence microscope (CKX53, Olympus Corporation, Tokyo Japan) under appropriate excitation/emission (MitoSOX^TM^ Red with Ex/Em = 510/580 and DCFH with Ex/Em= 495/529).

### Statistics

All data were analyzed using 2- or 3-way ANOVA with HS exposure, inhibitor treatment, and/or recovery time as classified factors. When main effects were significant, group differences were evaluated using t-test. In cases of significant interactions, Turkey’s post hoc test was applied for multiple comparisons. Analyses were based on 3 independent culture experiments, in which 3 replicate cultures of each cell type and treatment were performed. Details of the 2- or 3-way ANOVA analyses were included in the Supplementary results. Data are presented as means ± SE and differences were considered statistically significant at *P* < 0.05. Statistical analyses were performed by SAS enterprise guide 8.3 (64-bits).

## Results

### Heat stress impairs cell viability and alters lipid contents in granulosa and theca cells

**Fig. 1:**
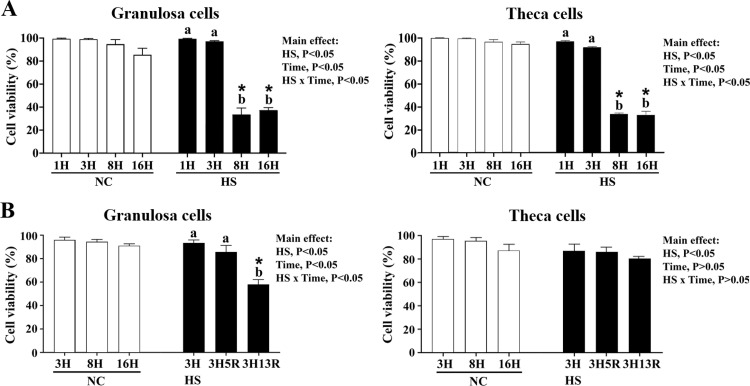
**Heat exposure induces cell death of chicken ovarian granulosa and theca cells.** Chicken ovarian granulosa and theca cells (from F2-F5 follicles) grown to 85 % confluence were heat-stressed (HS, 42°C) for various durations (1, 3, 8, or 16 hr; H) or maintained at 37°C as a neutral control (NC; panel A). In another round, cells were heat-stressed (HS, 42°C) for 3 hr (3H) and allowed recovery at 37°C for 5 (3H5R) or 13 hr (3H13R) (panel B). Cells collected at indicated time points were used for viability analysis. Results were analyzed by two-way ANOVA followed by post-hoc comparisons. *; significant effect by HS (*vs*. NC at the same time point), *P* < 0.05, *n* = 3. Means with different letters (a, b, c) within the same thermal treatment differ significantly among time points, *P* < 0.05, *n* = 3.

We first assessed the viability of hierarchical GCs and TH cells under HS for various durations. In contrast to cells at 37°C (NC), cells cultured at 42°C for 8 hr (HS8H) or longer exhibited significantly lower viability in both cell types, and interestingly, HS for 16 hr (HS16H) did not further exacerbated cell death (*P* < 0.05, Fig. 1, panel A). Following acute HS for 3 hr (HS3H), GCs showed impaired viability after 13 hr recovery (3H13R)(95 % vs. 60 %, *P* < 0.05, Fig. 1, panel B), while TH cells were resistant and remained no significant changes (90 % vs. 80 %). These results are consistent with previous studies showing HS-induced mild apoptosis in chicken GCs exposed to 41-42 °C for prolonged amounts of time (Yang et al., 2021; Yan et al., 2022) and suggest a higher stress tolerance for TH cells than GCs (Tilly et al., 1991a; 1991b). Cells heat-stressed at 42°C for in vitro studies also coincided with the physiological response as acute HS increased rectal temperature to ∼42–43°C in laying hens (Rozenboim et al., 2007; Cheng et al., 2018a; 2018b). Our pilot results suggested that HS above 43°C caused massive cell death, making the temperatures unsuitable for mechanistic studies (data not shown).

**Fig. 2:**
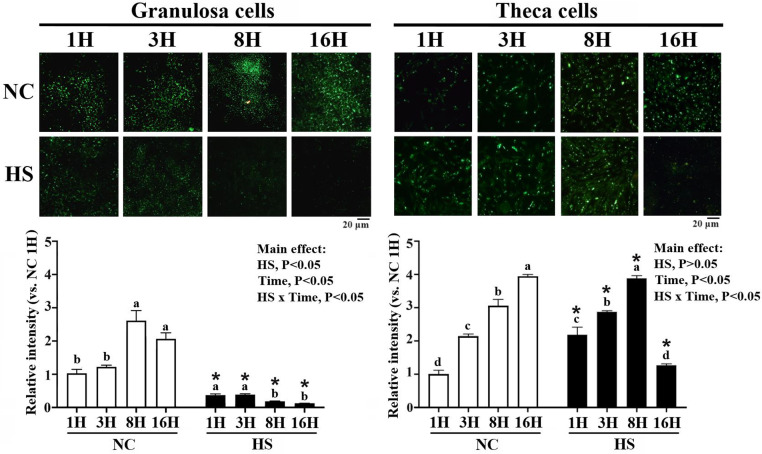
**Effects of heat stress on lipid droplet accumulation of granulosa and theca cells.** Granulosa and theca cells at 85 % confluence were heat-stressed (HS, 42°C) for various durations (1, 3, 8, or 16 hr; H) or maintained at 37°C as a neutral control (NC). Cells collected at indicated time points were used for neutral lipid droplet staining by BODIPY^TM^493/503. *; significant effect by HS (*vs*. NC at the same time point), *P* < 0.05, *n* = 3. Means with different letters (a, b, c) within the same thermal treatment differ significantly among time points, *P* < 0.05, *n* = 3. BODIPY^TM^493/503; 4,4-Difluoro-1,3,5,7,8-Pentamethyl-4-Bora-3a,4a-Diaza-s-Indacene.

Neutral lipid accumulation assessed by BODIPY staining was visualized after 1, 3, 8 and 16 h in culture and expressed as intensities relative to staining in 1H NC cultures of each cell type. Results showed a time-dependent increase of lipid droplets (LDs) in both GC and TH cells cultured at NC (*P* < 0.05, Fig. 2), suggesting a surplus energy status for de novo lipid synthesis (Olzmann and Carvalho, 2019). Heat stress prevented LD accumulation in GC as early at 1H, and continued to decline as the duration of HS prolonged to 8H and 16H (Fig. 2) in coincidence with the overtly impaired cell viability (Fig. 1. panel A). In contrast, the relative intensity of LD staining significantly increased in TH cells following HS at 1H (2-fold), 3H (3-fold), and 8H (4-fold), but rapidly diminished at 16H even lower than those at HS1H and similar to those of 1H—NC cells (*P* < 0.05, Fig. 2). The totally opposite alterations of cellular neutral lipid reservoir between GCs and TH cells manifest differential energy/nutrient need in adaption to HS and during subsequent recovery and thus may account for their disparate thermotolerance. Besides, the rapid alterations of LDs in TH cells under prolonged HS particularly from 8H to 16H suggest a rapid metabolic adaption in lipid dynamics including early de novo synthesis, followed by disposal through mobilization into export routes, and even via turnover such as dissipation by mitochondrial β-oxidation, or sequestration and conversion into other glycerophospholipids (Solano et al., 2025).

### Heat stress upregulates ApoB expression and MTTP activity to potentiate VLDL secretion

**Fig. 3:**
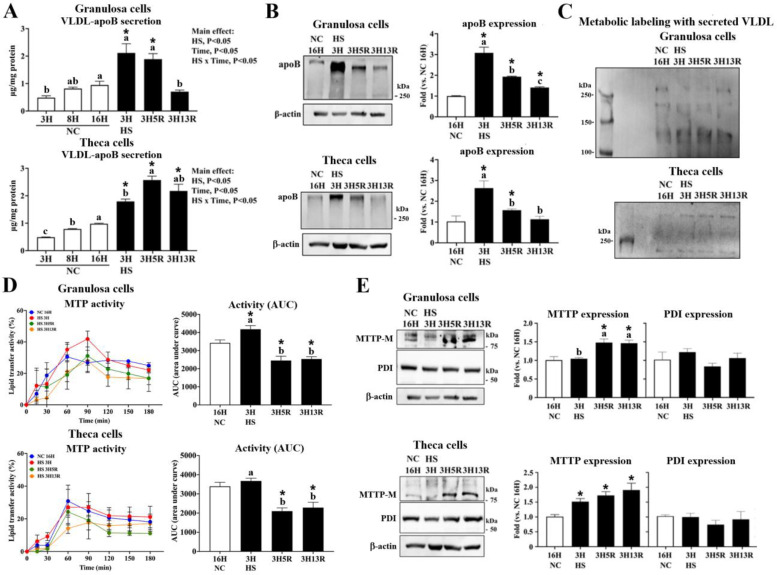
**Granulosa and theca cells respond to heat stress for VLDL-apoB secretion.** Granulosa and theca cells were heat-stressed (HS, 42°C) for 3 hr (3H) and allowed recovery at 37°C for 5 (3H5R) or 13 hr (3H13R). Cells maintained at 37 °C served as a neutral control (NC). Medium collected at indicated time points was used for VLDL isolation. VLDL-apoB was determined by ELISA in isolated VLDL (panel A) and collected cells were used for apoB (panel B), MTTP-M (microsomal triglyceride transfer protein subunit M), and PDI (protein disulfide isomerase; MTTP-P subunit) expression by Western blotting (panel E), and for MTTP activity analysis (panel D). In metabolic labeling studies (panel C), cells were pre-treated with azidohomoalanine (AHA, a methionine analogue, 50 μM) overnight and then heat-stressed at 42°C for 3 hr and allowed recovery at 37°C. Medium were collected for VLDL isolation. The newly synthesized proteins identified by AHA incorporation in VLDL extracts were labeled with biotin azide and analyzed by regular Western blotting using streptavidin-HRP conjugate as a probe. Results of Western blot were normalized to β-actin and expressed as ratios relative to the control (NC 16H). *; significant effect by HS (*vs*. NC at the same time point in panel A, or *vs*. NC 16H in panel B, D, and E), *P* < 0.05, *n* = 3. Means with different letters within the same thermal treatment (a, b, c) differ significantly among time points, *P* < 0.05, *n* = 3.

We then examined molecular mechanisms of VLDL production operative during HS and following recovery. Consistent with LD outcomes (Fig. 2), both GC and TH cells cultured at NC exhibited a time-dependent increase of VLDL-apoB secretion (*P* < 0.05, Fig. 3, panel A). Acute HS for 3 h (HS3H) significantly enhanced VLDL-apoB secretion, apoB expression, MTTP protein abundance, and particularly MTTP activity in GCs (*P* < 0.05, Fig. 3, panel A, B, D, E). Metabolic labeling studies for de novo apoB synthesis and secretion further supported that the apoB moiety in secreted VLDL is derived from de novo synthesis (Fig. 3, panel C). Following HS3H, both types of cells exhibited a decline of apoB expression and MTTP activity but MTTP-M expression increased, and PDI, the P-subunit of MTTP, remained no changes at HS3H and during recovery (3H5R to 3H13R) (*P* < 0.05, Fig. 3, panel B, D, E), suggesting that apoB availability and other effectors by HS induction are also involved in MTTP activity in addition to MTTP protein abundance *per se* (Liao et al., 2003).

Following HS3H, VLDL-apoB secretion by GCs declined significantly during the recovery period (3H5R to 3H13R) to a level even lower than NC-cells at 16H, while TH cells showed a significant increase at 3H5R and remained at higher levels than those of NC-cells (*P* < 0.05, Fig. 3, panel A). In metabolic labeling studies, however, the intensity of AHA-labeled VLDL-apoB fragments was sustained at 3H13R in both cell types (Fig. 3, panel C), conflicting to the decline of VLDL-apoB secretion of GCs during recovery period. This discrepancy can be attributed the incorporation of methionine analogue AHA into the apoB moiety in secreted VLDL, which impairs apoB recognition by VLDL receptors, leading to accumulation in the medium. As a result, the significant reduction VLDL-apoB secretion at 3H13R in GCs in couple with suppressed LDs under HS (Fig. 2) may indicate re-uptake of VLDL by GCs themselves due to energy and nutrient demands during post-stress recovery (Cheng et al., 2018a; 2018b). As evidenced by the significantly impaired GC viability at 3H13R, but not in TH cells, this nutrient supply is more critical to GCs, while TH cells likely surpass in metabolic adaptions and thus sustain VLDL secretion and viability.

### Lomitapide and Mipomersen suppress VLDL secretion

**Fig. 4:**
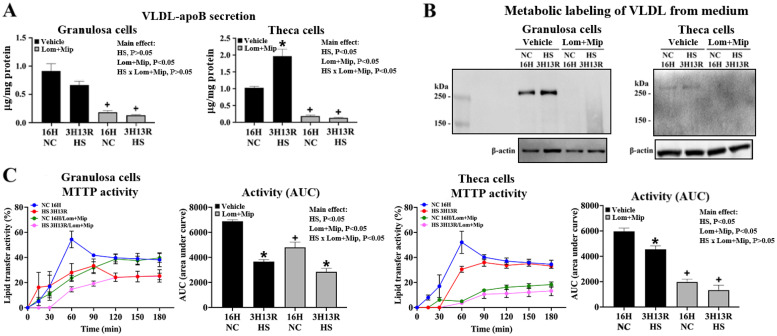
**Effects of Lomitapide and Mipomersen on VLDL secretion of granulosa and theca cells under heat stress.** Granulosa and theca cells were treated with Lomitapide (Lom) and Mipomersen (Mip) (MTTP and ApoB inhibitor, respectively, 2.5 μM for each) for 2 hr. After washout, cells were heat-stressed (HS, 42°C) for 3 hr (3H) and allowed recovery at 37°C for 13 hr (3H13R). Cells maintained at 37°C served as a neutral control (16H NC). Medium were collected for VLDL isolation and then used for VLDL-apoB determination through the ELISA method (panel A) and cells were used for MTTP activity analysis (panel C). In metabolic labeling studies (panel B), cells pre-treated with azidohomoalanine (AHA, 50 μM) overnight and then with Lom and Mip for 2 hr were subjected to HS treatment at 42°C for 3 hr and allowed recovery at 37°C. Medium were collected for VLDL isolation. AHA incorporation in VLDL protein extracts was labeled with biotin azide and analyzed by the regular Western blot method. *; significant effect by HS (*vs*. NC 16H within the same pharmacological treatment), +; significant effect by Lom+Mip (*vs*. vehicle within the same thermal treatment at the same time), *P* < 0.05, *n* = 3.

Treatment of Lom and Mip significantly suppressed VLDL-apoB secretion and MTTP activity in both cell types regardless of heat exposure and recovery (*P* < 0.05, Fig. 4, panel A to C). The suppressive effects were more pronounced in TH cells particularly at 3H13R (Fig. 4, panel A, C), suggesting a more active regulatory interplay with other stress factors, such as heat shock proteins (HSPs), known to modulate MTTP activity for cargo lipid export via a post-translational loop (Hussain et al., 2003; Liao et al., 2003).

### Inhibition of VLDL secretion differentially rescues cell viability under prolonged HS

**Fig. 5:**
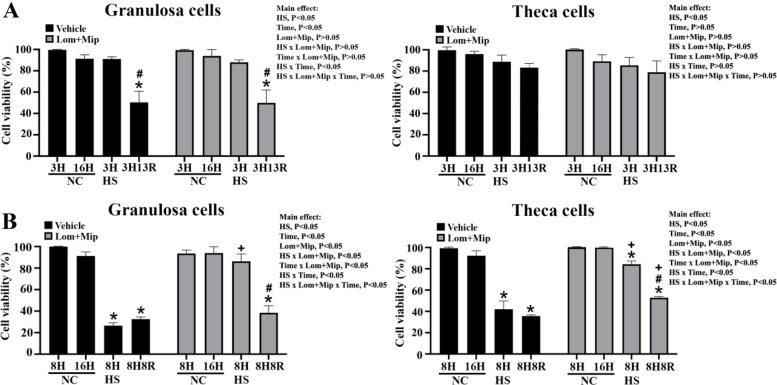
**Effects of Lomitapide and Mipomersen on granulosa and theca cell viability under heat stress.** Granulosa and theca cells were treated with Lomitapide (Lom) and Mipomersen (Mip) (2.5 μM for each) for 2 hr. After washout, cells were heat-stressed (HS, 42°C) for 3 (3H, panel A) or 8 hr (8H, panel B) and allowed recovery for 13 (3H13R) or 8 hr (8H8R), respectively. Cells collected at indicated time points were used for viability analysis. *; significant effect by HS (*vs*. NC within the same pharmacological treatment at the same time point), #; significant effect by time (*vs*. 3H or 8H within the same thermal and pharmacological treatment), +; significant effect by Lom+Mip (*vs*. vehicle of the same thermal treatment at the same time), *P* < 0.05, *n* = 3.

In accordance with the results in previous studies (Chen et al., 2025), treatment with Lom and Mip to block VLDL secretion failed to affect cell viability in both cell types cultured at NC (Fig. 5, panel A). Surprisingly, Lom and Mip improved GC viability at HS8H but not after recovery for 8 hr (8H8R) or at 3H13R, and rescued TH cell viability at HS8H and 8H8R (*P* < 0.05, Fig. 5, panel B), suggesting that enhanced VLDL secretion mediates, at least in part or transiently, the detrimental effects of HS on cell viability, rather than acts as a defensive response to protect the cells. The suppressive effect by HS on cell viability is more pronounced in GCs regardless of HS duration and recovery, while TH cells sustained VLDL secretion during recovery (Fig. 3, panel A) and exhibited more rapid dissipation of LDs under prolonged HS8H to HS16 (Fig. 2) and improved cell viability in the presence of Lom and Mip (Fig. 5), linking the dynamics of cellular lipids with thermotolerance.

### Inhibition of VLDL secretion differentially affects lipid dynamics

**Fig. 6:**
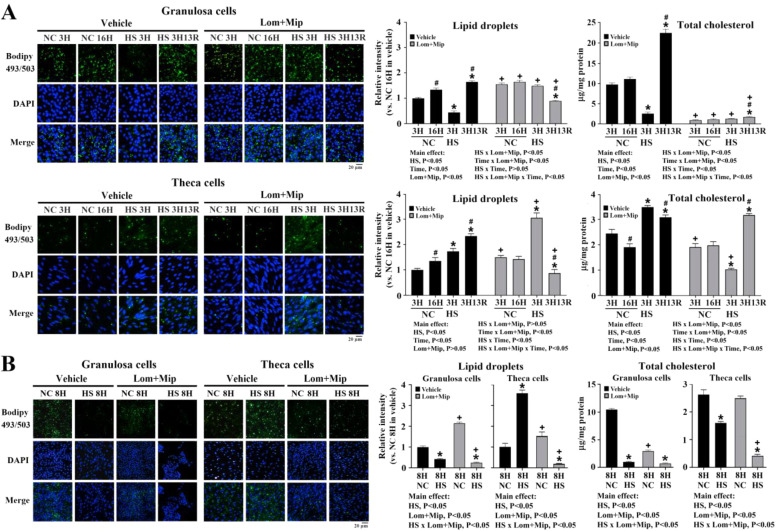
**Effects of Lomitapide and Mipomersen on neutral lipid and cholesterol content of granulosa and theca cells under heat stress.** Granulosa and theca cells were treated with Lomitapide (Lom) and Mipomersen (Mip) (2.5 μM for each) for 2 hr. After washout, cells were heat-stressed (HS, 42°C) for 3 (3H, panel A) or 8 hr (8H, panel B) and allowed recovery for 13 (3H13R) or 8 hr (8H8R), respectively. Cells collected at indicated time points were used for neutral lipid staining by BODIPY^TM^493/503 and for cholesterol content determination using commercial kits. Results were analyzed by two-way or three-way ANOVA followed by post-hoc comparisons. *; significant effect by HS (*vs*. NC within the same pharmacological treatment at the same time), #; significant effect by time (*vs*. 3H of the same thermal and pharmacological treatment), +; significant effect by Lom+Mip (*vs.* vehicle of the same thermal treatment at the same time), *P* < 0.05, *n* = 3.

In consistence with the results in Fig. 2, LDs accumulated along time course in both GC and TH cells cultured at NC, and both acute (HS3H) and prolonged HS (HS8H) suppressed LDs in GCs, but enhanced LD accumulation in TH cells (*P* < 0.05, Fig. 6, panel A, B). In contrast to NC-cells at 16H and those immediately at HS3H, recovery for 13 hr (3H13R) resulted in rapid accumulation of LDs, particularly in GCs (*P* < 0.05, Fig. 6, panel A), partially due to re-uptake of secreted VLDL (Fig. 3, panel A). Under NC, Lom and Mip promoted LDs at 3H, 8H, and 16H in GCs and at 3H and 8H in TH cells, but eliminated the time-dependent increase of LDs from 3H to 16H in both cell types (*P* < 0.05, Fig. 6, panel A). Under HS conditions, Lom and Mip enhanced LDs at HS3H in both cell types, but surprisingly suppressed LDs at 3H13R and HS8H, reversing the increase of LDs during recovery (HS3H to 3H13R) to a decline fashion (*P* < 0.05, Fig. 6, panel A, B). The suppressive effect and decline were more profound in TH cells.

Under NC, TH cells showed a slight (3H to 16H) decrease of cholesterol content, while GCs sustained stable levels (*P* < 0.05, Fig. 6, panel A). Cellular cholesterol contents were greatly suppressed at HS3H and HS8H in GCs, but potentiated at HS3H and decreased at HS8H in TH cells (*P* < 0.05, Fig. 6, panel A, B). Recovery for 13 hr (3H13R) following HS3H increased cholesterol content in both cell types in contrast to those at NC16H and HS3H, while this increase was more pronounced in GCs than TH cells (*P* < 0.05, Fig. 6, panel A). Depletion of cholesterol reservoir has been shown to abate cellular structural integrity and diminish thermotolerance (Kanamori et al., 2025). Accordingly, the remarkable suppression of cholesterol at HS3H and later replenishment in couple with significantly impaired cell viability at 3H13R in GCs (Fig. 6, panel A) suggest a sluggish metabolic adaption in lipid dynamics to sustain membrane integrity and thus are susceptible to structural demolition upon heat exposure.

**Fig. 7:**
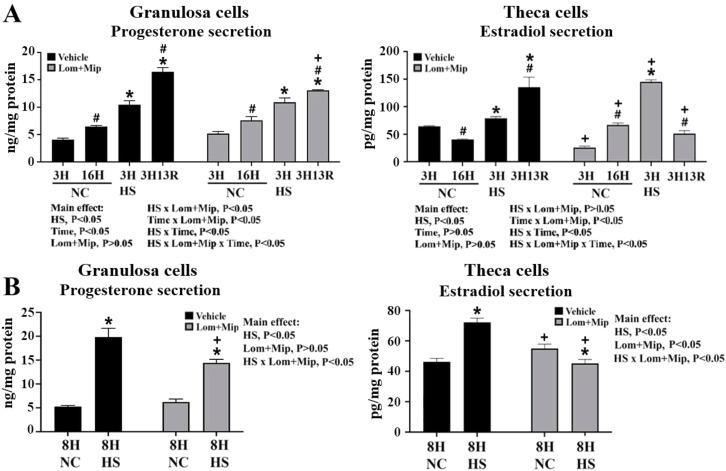
**Effects of Lomitapide and Mipomersen on sex steroid secretion of granulosa and theca cells under heat stress.** Granulosa and theca cells were treated with Lomitapide (Lom) and Mipomersen (Mip) (2.5 μM for each) for 2 hr. After washout, cells were heat-stressed (HS, 42°C) for 3 (3H, panel A) or 8 hr (8H, panel B) and allowed recovery for 13 (3H13R) or 8 hr (8H8R), respectively. Medium collected at indicated time points were used for progesterone (P4) and estradiol (E2) determination through ELISA kits. *; significant effect by HS (*vs*. NC within the same pharmacological treatment at the same time), #; significant effect by time (*vs*. 3H within the same thermal and pharmacological treatment), +; significant effect by Lom+Mip (*vs.* vehicle within the same thermal treatment at the same time), *P* < 0.05, *n* = 3.

In contrast to increased accumulation of LDs, surprisingly, Lom and Mip greatly depressed cholesterol contents in GCs regardless of NC or HS and recovery, while TH cells only declined at NC3H, HS3H, and HS8H, but remained no changes at NC8H and NC16H, and even elevated at 3H13R (*P* < 0.05, Fig. 6, panel A, B). Lom and Mip also reversed the decrease of cholesterol content during recovery (HS3H to 3H13R) to an increase fashion in both types of cells with a more profound rebound in TH cells to reach a level comparable to the vehicle control at 3H13R (*P* < 0.05, Fig. 6, panel A). The depressed cholesterol reservoir by Lom and Mip can be attributed to the deficiency of de novo synthesis as genetic ablation of hepatic MTTP or pharmacological inhibition on its activity downregulates hepatic HMG-CoAR (3‑hydroxy-3-methylglutaryl-CoA reductase) expression in mice (Dikkers et al., 2014). Besides, continuous sex steroids may further deplete cellular cholesterol reservoir. As assessed by P4 and E2 production, GCs and TH cells showed a 10-fold difference in steroidogenic activity (Fig. 7, panel A) and thus GCs may experience a more severe shortage of cholesterol for membranous structure maintenance and repairment under HS. Since the suppressive effect of Lom and Mip on VLDL-apoB secretion was more pronounced in TH cells than GCs (Fig. 4, panel A), the rapid dissipation of LDs and replenishment of cholesterol from HS3H to 3H13R in TH cells (Fig. 6, panel A) excludes the causal infectiveness due to drug metabolism, instead likely operated via a timely intrinsic mechanism for dynamic lipidomic remodeling to sustain membrane integrity. Despite greatly depleting cellular cholesterol reservoir at HS8H, Lom and Mip still rescued TH cell viability at HS8H and 8H8R but only at HS8H in GCs (Fig. 5, panel B), suggesting that in addition to lipid dynamics, enhanced VLDL production by HS activates other machineries to potentiate cell death, likely via the later apoptotic program after heat shock (Kanamori et al., 2025), while TH cells are more resistant to the apoptotic development.

### Inhibition of VLDL secretion upregulates PCNA and IL-1β expressions and differentially affects steroidogenesis

Similar to P4 and E2 production along follicle advancement within the hierarchy (Bahr et al., 1983; Johnson, 2000), GCs cultured at NC exhibited an increase of P4 production while E2 secretion by TH cells decreased along the time course (3H to 16H) (*P* < 0.05, Fig. 7, panel A). Heat stress promoted P4 production and E2 secretion regardless of HS durations and recovery and resulted in an increasing fashion of E2 secretion (*P* < 0.05, Fig. 7, panel A, B), suggesting that HS enforces GC differentiation but hampers the maturation of TH cells. Increased sex steroid outputs at prolonged HS8H in both cell types (Fig. 7, panel B) may contribute to the depletion of cell cholesterol reservoirs (Fig. 6, panel B) and thus potentiate cell death (Fig. 5, panel B).

Lom and Mip had no effects on P4 production in GCs cultured at NC or immediately at HS3H, but suppressed P4 production at HS8H and 3H13R (*P* < 0.05, Fig. 7, panel A). In TH cells, E2 secretion were diminished by Lom and Mip at NC3H but increased later at NC8H and NC16H, and also increased at HS3H but declined after 3H13R and at prolonged HS8H (*P* < 0.05, Fig. 7, panel A, B). The increase of E2 secretion from HS3H to 3H13R was reversed by Lom and Mip leading to a decline fashion (*P* < 0.05, Fig. 7, panel A). Since cells were isolated from the F2 to F5 stage follicles and may retain their intrinsic steroidogenic patterns as shown by increasing P4 production and diminishing E2 secretion along the time course under NC (Fig. 7, panel A), the altered P4 and E2 competence and secretory fashion by HS and/or Lom and Mip thus indicates interfered cell fate alone follicle advancement toward maturation (Bahr et al., 1983). Accordingly, increased P4 and E2 production by HS suggest imposed GC differentiation but interfered TH cell maturation. Intervention of VLDL production decelerates GC maturation under HS and TH cell differentiation under NC and acute HS, but potentiates TH cell maturation after recovery and under prolonged HS.

StAR expressions were upregulated at HS3H, HS8H and post-recovery at 3H13R in GCs, and at HS8H but decreased at 3H13R in TH cells (*P* < 0.05, Fig. S1, panel A, B). Lom and Mip suppressed StAR expressions in both cell types cultured at NC and at 3H13R in GCs and HS8H in TH cells, but upregulated StAR expression at HS8H in GCs and at HS3H and 3H13R in TH cells (*P* < 0.05, Fig. S1, panel A, B). Since avian follicle steroid intermediates are shuttled mutually among GC, TH externa, and interna layer for P4, E2, and testosterone synthesis, respectively (Porter et al., 1989; Johnson, 2000), the results of StAR expressions thus may merely reflect its role to mediate cholesterol traffics to mitochondrial steroidogenesis, not accounting for final outputs of sex steroids.

**Fig. 8:**
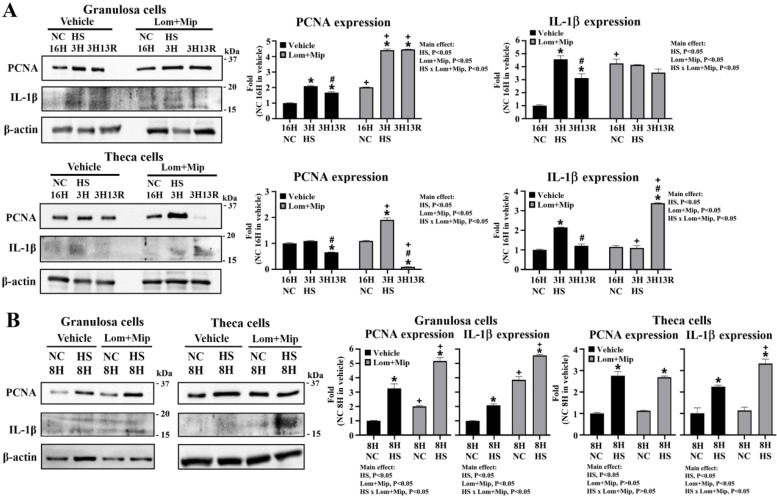
**Effects of Lomitapide and Mipomersen on cell proliferation and inflammatory response of granulosa and theca cells under heat stress.** Granulosa and theca cells were treated with Lomitapide (Lom) and Mipomersen (Mip) (2.5 μM for each) for 2 hr. After washout, cells were heat-stressed (HS, 42°C) for 3 (3H, panel A) or 8 hr (8H, panel B) and allowed recovery for 13 (3H13R) or 8 hr (8H8R), respectively. Cells collected at indicated time points were used for PCNA (proliferating cell nuclear antigen) and IL-1β (interleukin-1β) expression through Western blot analysis. Results were normalized to β-actin and expressed as ratios relative to the control (NC 16H or NC 8H). *; significant effect by HS (*vs*. NC 16H or NC 8H within the same pharmacological treatment), #; significant effect by time (*vs*. 3H within the same thermal and pharmacological treatment), +; significant effect by Lom+Mip (*vs.* vehicle of the same thermal treatment at the same time), *P* < 0.05, *n* = 3.

Heat stress upregulated PCNA and IL-1β expressions at HS3H, 3H13R and HS8H in GCs, while in TH cells PCNA expression was downregulated at 3H13R but upregulated at HS8H, and IL-1β was upregulated at HS3H and HS8H (*P* < 0.05, Fig. 8, panel A, B). Both GC and TH cells showed a decline of PCNA and IL-1β expression during recovery to 3H13R (*P* < 0.05, Fig. 8, panel A). Lom and Mip upregulated PCNA and IL-1β expressions in GCs cultured at NC, but had no effects on TH cells (*P* < 0.05, Fig. 8, panel A, B). Under HS conditions, Lom and Mip promoted PCNA expressions at HS3H, 3H13R, and HS8H, and IL-1β expressions at HS8H in GCs, while in TH cells PCNA was upregulated at HS3H but decreased at 3H13R and IL-1β was downregulated at HS3H but increased at 3H13R and HS8H (*P* < 0.05, Fig. 8, panel A, B). During recovery from HS3H to 3H13R, Lom and Mip abolished the decline of PCNA and IL-1β expressions in GCs, but amplified the decrease of PCNA, and even reversed IL-1β expressions to an increasing fashion (*P* < 0.05, Fig. 8, panel A). Cytokines such as tumor necrosis factor-α (TNF-α) and IL-1β have been shown to govern the transition of follicular cell proliferation to differentiation, and apoptosis (Brännström, 2004; Silva et al., 2020). The differentially upregulated PCNA and IL-1β expressions thus mediate HS and/or Lom and Mip impacts on follicular cell fate alone maturation.

### Inhibition of VLDL secretion differentially regulates redox status

**Fig. 9:**
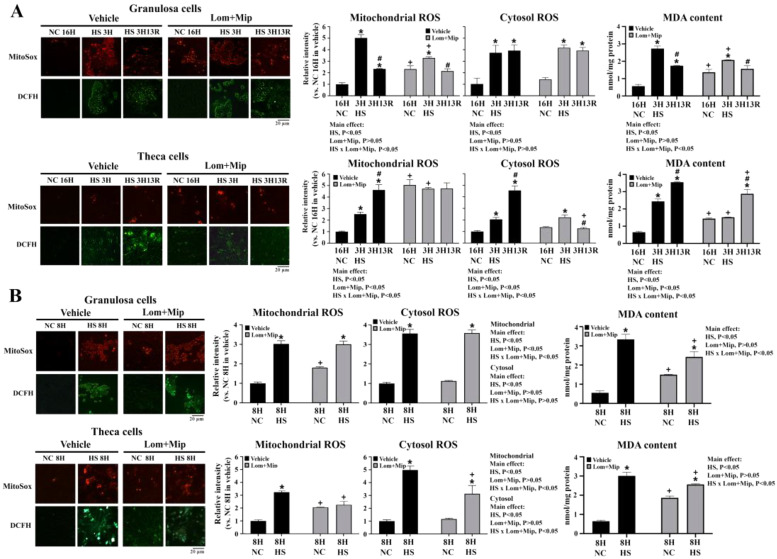
**Effects of Lomitapide and Mipomersen on ROS production and MDA content of granulosa and theca cells under heat stress.** Granulosa and theca cells were treated with Lomitapide (Lom) and Mipomersen (Mip) (2.5 μM for each) for 2 hr. After wash out, cells were heat-stressed (HS, 42°C) for 3 (3H, panel A) or 8 hr (8H, panel B) and allowed recovery for 13 (3H13R) or 8 hr (8H8R), respectively. Cells collected at indicated time points were used for MDA (malondialdehyde) content determination and ROS production by MitoSOX (mitochondrial) or DCFH (2,7-Dichlorodihydrofluorescein diacetate)(cytosolic) staining. *; significant effect by HS (*vs*. NC 16H or NC 8H within the same pharmacological treatment), #; significant effect by time (*vs*. 3H with the same thermal and pharmacological treatment), +; significant effect by Lom+Mip (*vs.* vehicle within the same thermal treatment at the same time), *P* < 0.05, *n* = 3.

Heat stress significantly provoked ROS production from mitochondrial and cytosol origin as shown by MitoSOX and DCFH staining, respectively, and caused MDA accumulation in both cell types regardless of HS duration and recovery (*P* < 0.05, Fig. 9, panel A, B). Recovery for 13 hr (3H13R) resulted in a decline of mitochondrial ROS generation and MDA accumulation in GCs, but surprisingly augmented the oxidative insults in TH cells (*P* < 0.05, Fig. 9, panel A). Despite the increased oxidative insults during recovery, TH cells sustained viability at 3H13R (Fig. 5, panel A), thus manifest higher oxidative stress tolerance.

Under NC, Lom and Mip increased mitochondrial ROS production in both cell types (*P* < 0.05, Fig. 9, panel A, B). In GCs, Lom and Mip relieved mitochondrial ROS production immediately at HS3H but not at 3H13R and HS8H, whereas in TH cells mitochondrial ROS production was significantly exacerbated at HS3H but alleviated at HS8H, and cytosolic ROS was alleviated at 3H13R and HS8H (*P* < 0.05, Fig. 9, panel A, B). Lom and Mip abolished the increase of mitochondrial ROS production from HS3H to 3H13R in TH cells and reversed the increase of cytosolic ROS to a declining fashion (*P* < 0.05, Fig. 9, panel A). Interestingly, Lom and Mip promoted cellular MDA accumulation in both GC and TH cell cultured at NC, but relieved MDA accumulation at HS3H, HS8H, and/or 3H13R (*P* < 0.05, Fig. 9, panel A, B). In contrast to GCs, TH cells showed more remarkable increases in mitochondrial and cytosolic ROS production from HS3H to 3H13R and at HS8H, indicating more electron leakages likely due to a higher mitochondrial oxidative phosphorylation for ATP yield in adaption to HS (Cheng et al., 2018a; 2018b; Olzmann and Carvalho, 2019). Besides, the effect by Lom and Mip on mitochondrial ROS release was dramatically upregulated at HS3H and sustained to 3H13R in TH cells, whereas GCs responded with a mild increase but progressed into a decline fashion. In contrast to those in GCs, the decline of cytosolic ROS from HS3H to 3H13R and at HS8H by Lom and Mip in TH cells suggest a more active proteostatic machinery to decrease or quench cytosolic ROS produced from NADH oxidase, P 450 cytochromes, peroxisomes, and/or ER (Cao and Kaufman, 2014; Shu et al., 2023).

## Discussion

For the first time, the present results demonstrated that HS impairs chicken follicular cell viability by operating at cargo lipid export via VLDL output, in which GC and TH cells exhibit disparate responses in lipid dynamics; persistently suppressed cellular neutral lipids and cholesterol in GCs, while TH cells accumulate neutral lipids but rapidly dissipate them after prolonged to HS8H or HS16H. These alterations tightly coincide with the occurrence of overt cell death after HS exposure and with improved viability by Lom and Mip, and thereby confirm the salient thermotolerant in TH cells than GCs. Acute HS3H promoted VLDL secretion in both cell types but only GCs showed re-uptake of VLDL secretion and impaired viability after recovery at 3H13R. Under interfered VLDL secretion, TH cells showed persistently relieved ROS provocation and MDA accumulation and sustained and/or improved viability in couple with lipid dynamics; timely depletion of LDs and replenishment of cholesterol during HS3H to 3H13R and even alleviated neutral lipid accumulation at prolonged HS8H, suggesting a highly adaptive machinery for lipid disposal via VLDL export, dissipation by mitochondrial β-oxidation, and/or turnover into glycerophospholipids and sphingolipids (Solano et al., 2025). The altered steroidogenic competences by HS suggest enforced GC differentiation but retarded maturation in TH cells. Intervention of VLDL production decelerates the maturation of GCs under HS and during recovery and TH cells under NC and acute HS, but facilitates TH cell maturation during recovery and under prolonged HS.

In our recent report (Dettipponpong et al., 2025), inhibition of VLDL secretion by Lom and Mip significantly alleviated ER stress and protein ubiquitinylation in small yellow follicles (SYFs) under cultures regardless of NC or HS and recovery, but exacerbated TAG accumulation, protein carbonylation, and MDA accumulation. Lom and Mip also enhanced cell apoptosis in SYFs cultured at NC, but had no such effects under HS despite transiently worsened cell proliferation and E2 secretion, suggesting that routine secretion of VLDL by SYFs is necessary to sustain cell viability and support SYF development, while under HS, this machinery provisionally rescues steroidogenesis and cell proliferation. In contrast to SYFs, VLDL production mediates, at least in part, the detrimental effect of HS on hierarchical follicle cell survival and maturation, and is necessary to support cell maturation under normal conditions. These results manifest the role of ovarian VLDL production in follicle development in a stage and stress-dependent manner. Nevertheless, additional mechanistic studies involving lipidomic dynamics, membrane integrity, apoptotic cascades, ER and proteostatic stress, and autophagic process are required to elucidate the interplay of VLDL production in HS-induced follicular cell death.

In laying stage, approximately 30 % of circulating VLDL are targeted to the ovarian follicles for yolk deposition (Bacon et al., 1978) and laying hen GCs possess a much higher LPL (lipoprotein lipase) activity than the adjacent TH cells, up to half of that in adipose tissues (Benson et al., 1975). The presence of functional VLDL production in chicken ovarian tissues therefore may serve as an evolutionarily conserved machinery to maintain cellular lipid homeostasis against lipid overload and following lipotoxicity to escort follicle development as suggested by the failing human heart (Borén et al., 1998). In mammals, the necessity of follicular lipid homeostasis to sustain mitochondrial functions such as bioenergetics and steroidogenesis, regulate redox status and cell fate, and support oocyte maturation have been documented to secure follicle development (Leroy et al., 2005; Aardema et al., 2011; Bertevello et al., 2018). As shown by the time-dependent increase of VLDL secretion along cellular LDs and/or cholesterol accumulation in both cell types under NC, intervention of VLDL secretion promoted LD accumulation, provoked ROS production and MDA contents, differentially upregulated PCNA and IL-1β expressions, and affected E2 production leading to altered secretion fashion. The mechanisms of impaired steroidogenesis due to lipid accumulation have been shown in goose GCs overexpressing fatty acid synthase (Chen et al., 2020). In broiler breeder hens, excess ovarian lipid accumulation and resultant lipotoxicity have been linked to impaired secretion of P4 and matrix metalloproteinases for follicle wall rupture, inflammation as shown by increased leukocyte infiltration and IL-1β production (Pan et al., 2012; Xie et al., 2012; Liu et al., 2014), and a higher degree of GC apoptosis (Xie et al., 2012; Liu et al., 2016), which in turn provoke follicle atresia and retard follicle growth, maturation, and ovulatory process, ultimately leading to poor egg production (Chen et al., 2006; Li et al., 2020).

In mammals, hepatic apoB expression is primarily regulated at co- and post-translational level involving with HSPs, MTTP, and PDI for chaperoning and targeting for degradation in the proteosomes (Gusarova et al., 2001; Hussain et al., 2003). In addition, hepatic apoB mRNA expression has been shown to respond to estradiol induction in birds (Kirchgessner et al., 1987). Accordingly, the upregulation of apoB protein at HS3H may be attributed to increased apoB gene transcription due to the rise of estradiol simultaneously induced by HS, while following upregulated HSP25 and HSP70 expressions account for the decline of apoB protein abundance during recovery (Cheng et al., 2018a). Our previous studies showed that apoB mRNA transcripts were upregulated by HS and lasted during recovery in chicken small yellow follicles (Dettipponpong et al., 2025). In oviparous *Caenorhabditis elegans*, HSF-1 (heat shock factor 1), a master transcriptional factor by HS induction to prime regulatory gene expressions such as HSPs (Gomez-Pastor et al., 2018), was shown to induce several gene expressions related to lipid metabolism and transport independently of HS, including *vit-1, −3, −4, −5* (vitellogenin, the distant homologue of mammalian apoB) for lipid transport and *fat-7* (encoding Delta-9 desaturase) for monounsaturated fatty acid synthesis, while HS also upregulated *scp-1* (encoding SREBP cleavage-activating protein, SCAP) for cholesterol synthesis (Brunquell et al., 2016). These results again manifest the critical role of cargo lipid export in cellular lipid dynamics and moreover accentuate metabolic remodeling as a primitive adaption to HS across taxa, despite that the regulatory interplay of related genes in the ovary requires further validations.

Heat stress exerts detrimental effects mostly via ROS provocation, proinflammatory cytokines, and ER stress on ovarian functions in birds such as mitochondrial oxidative phosphorylation and steroidogenesis, cell proliferation, death, and follicle growth and atresia (Rozenboim et al., 2007; Li et al., 2020; Yang et al., 2021; Lin et al., 2022; Yan et al., 2022; Hu et al., 2025). Export of cellular cargo lipids by HS via VLDL delivery may collaterally output lipid-soluble vitamins such as tocopherol, and thus depletes its reservoir leading to increased oxidative stress (Terasawa et al., 1999). However, under normal conditions intervention of VLDL secretion increased mitochondrial ROS and MDA accumulation in both cell types, indicating enhanced mitochondrial β-oxidation in adaption to lipid accumulation. Most of cellular ROS are produced by mitochondrial electron leakage, while NADH oxidase, cytochrome P450, peroxisomes, and protein thiol redox regulation within the ER such as PDI-oxidoreductin (ERO)-1 and glutathione (GSH)/GSSG system contribute to cytosolic ROS accumulation (Cao and Kaufman, 2014; Shu et al., 2023). In mice, intervention of apoB synthesis by Mip alone was shown to trap lipids within the ER, which in turn triggers ER stress, autophagy and lysosomal lipolysis of TG to release FAs, followed by mitochondrial β-oxidation and ultimately prevented liver steatosis (Conlon et al., 2016). Metabolic adaptation in ATP supply contributes to thermal resistance (Hu et al., 2025; Kanamori et al., 2025). In contrast to GCs, TH cells showed rapid depletion of accumulated LDs from HS3H to 3H13R and HS8H to HS16H in the presence or absence of Mip and Lom, respectively, while sustained mitochondrial ROS production from HS3H to 3H13R, suggesting a timely metabolic adaption to mitigate energy stress by dissipating lipids through β-oxidation for a higher ATP yield instead of mostly via glycolysis/lactate pathway (Kanamori et al., 2025). Moreover, the remarkable alleviation of cytosolic ROS by Lom and Mip in TH cells may reflect a more efficient system to maintain protein-thiol redox homeostasis to relieve ER stress and following proteotoxicity, and thus contribute to the competence in cell viability under HS. Our proteomic studies demonstrated upregulation of ATP synthetase subunits, mitochondrial Complex I, and glycolytic enzymes in adaption to HS and during recovery (Cheng et al., 2018a, 2018b). These results thus manifest the vulnerable thermotolerance in GCs due to a high energy/nutrient demand and poor metabolic adaption (Jastrebski et al., 2017; Hu et al., 2025).

Cells mount a membrane stress response that senses lipid imbalance and rewire proteostasis, with cholesterol as a key stabilizer as depletion increases membrane fluidity and vulnerability under heat insults (Thibault et al., 2012; Kanamori et al., 2025). Cellular LDs cooperate with the ER and mitochondria to sequester FFAs and store TAG, buffering lipotoxicity, while hepatic TAG export via VLDL largely depends on phosphatidylcholine (PC) synthesis, when this is impaired, steatosis develops (Cole et al., 2012; Olzmann and Carvalho, 2019). A decrease of PC/PE (phosphatidylethanolamines) ratio impairs membrane integrity and causes steatohepatitis in mice (Li et al., 2006). During HS, cells undergo rapid lipidomic remodeling of phospholipids, acyl saturation, sphingolipids, and sterols that preserves membrane order and function to support thermotolerance (Thibault et al., 2012; Sun et al., 2024; Solano et al., 2025). It is well documented that HS activates sphingomyelinase to hydrolyze membrane sphingomyelin leading to ceramide generation and activation of downstream JNK and apoptotic signaling (Yabu et al., 2015). Our study aligns with the previous findings and expands on them by revealing that HS operates at VLDL production to affect cellular lipid dynamics, leading to structural damages and death even during recovery, particularly in GCs. In contrast, TH cells show greater resilience in thermoregulation even in the presence of Lom and Mip, as shown by rescued cell viability and sustained LDs and cholesterol reservoir under HS but rapid dissipation of LDs and cholesterol replenishment during recovery. Heat-induced cell death can be classified into early-phase necrosis, due to physical damages in structural integrity and late-phase apoptosis, driven by intrinsic signals such as ER stress and mitochondrial dysfunctions (Kanamori et al., 2025). The rapid lipidomic remodeling by promptly metabolic adaption thus may facilitate the superior thermotolerance and potentiate TH cell viability likely by operating at the early-phase necrosis under heat exposure, giving them a protective advantage over GCs. This function is crucial for maintaining follicular integrity and maturation, as TH layers produce collagen for structural support, as well as delivering nutrients, oxygen, and immune cells to the GC layer and oocyte (Magoffin, 2005; Young and McNeilly, 2010). Despite depressing cholesterol reservoir under prolonged HS, interference of VLDL production may operate at ER and mitochondrial stress to ease redox status and thereby alleviate late-phase apoptosis in both cell types (Kanamori et al., 2025)

Within the hierarchy, GCs differentiate into steroidogenic competence for P4 production along follicle advancement, while TH cells progressively degenerate in E2 production but engage in proliferative activity and protein synthesis for tissue remodeling (Bahr et al., 1983). Expression of PCNA is highest in prehierarchical GCs and declines as cells exit the cell cycle and acquire P4 competence, particularly into the hierarchy, making PCNA a sensitive readout of maturation status (Seol et al., 2006; Caicedo Rivas et al., 2016). Despite species and follicle size dependent, IL-1β and TNF-α govern the transition of follicular cell proliferation to differentiation and modulate follicle steroidogenesis and maturation (Brännström, 2004; Silva et al., 2020). In GCs, ceramide accumulation due to sphingomyelin hydrolysis was shown to mediate the inhibitory effect of IL-1β on steroidogenesis (Santana et al., 1996). These reports support the notion that IL-1β and PCNA integrate with lipid dynamics and related cues to govern cell differentiation and set steroidogenic competence during heat exposure. In vivo, HS suppressed serum P4 and E2 levels and reduced egg production with fewer hierarchical and SYFs (Rozenboim et al., 2007; Li et al., 2020), while in vitro studies with isolated cells or whole follicles showed enhanced P4 production but differentially altered E2 secretion depending on follicle class in association with impaired cell viability and proliferation, ROS and proinflammatory provocation, ER stress, and follicle atresia (Li et al., 2020; Yan et al., 2022; et al., Dettipponpong et al., 2025). Collectively, differentially upregulated PCNA and IL-1β expressions by HS in hierarchical follicles may alter secretory fashions of P4 and E2 and impose follicle maturation toward ovulation, while impair small follicle growth and steroidogenesis and even cause atresia, which afterwards limit the follicle pool and selection into the hierarchy, and thus less rapidly growing follicles, lower sex steroid outputs and circulating levels, leading to regressive ovarian functionality and thereby a decline of egg production.

## Conclusion

Heat stress employs in VLDL production of hierarchical follicles to augment its detrimental effects on follicular cell viability and maturation, while routine secretion of VLDL is necessary to sustain follicle development under normal conditions. TH cells are highly active in lipid dynamics and redox regulation in adaption to heat insults and thereby endowed with superior thermotolerance than GCs.

## Disclosures

The authors declare that they have no known competing financial interests or personal relationships that could have appeared to influence the work reported in this paper.

## Appendix Supplementary materials

Image, application 1
