# Supplementary material for: Heat stress enhances VLDL secretion in chicken ovarian follicles to potentiate its impact on follicular cell survival and maturation
Source: Poult Sci. 2025 Nov 19;105(1):106137. doi: 10.1016/j.psj.2025.106137 (PMC12723046; doi:10.1016/j.psj.2025.106137)
Supplement: Supplementary file 1 [file mmc1.docx]

**
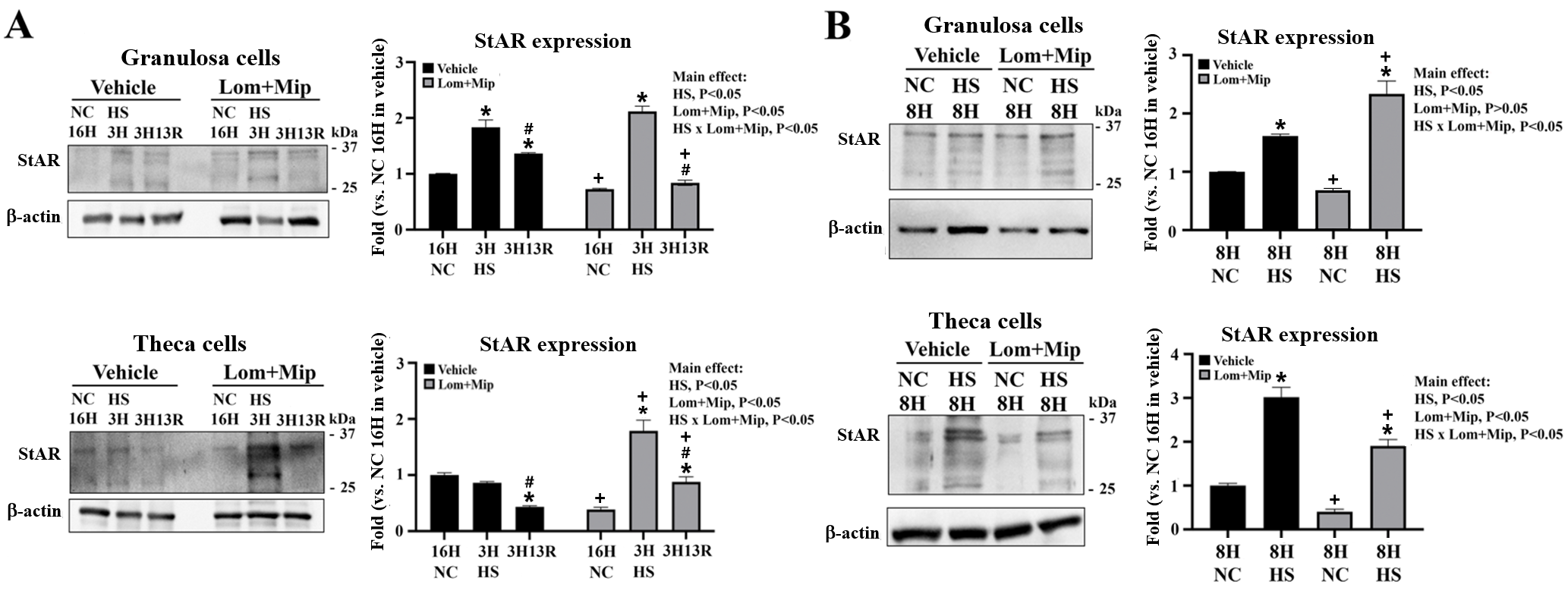
**

**Supplementary Fig. S1. Effects of Lomitapide and Mipomersen on StAR expression of granulosa and theca cells under heat stress.** Granulosa and theca cells were treated with Lomitapide (Lom) and Mipomersen (Mip) (2.5 μM for each) for 2 hr. After washout, cells were heat-stressed (HS, 42℃) for 3 (3H, panel A) or 8 hr (8H, panel B) and allowed recovery for 13 (3H13R) or 8 hr (8H8R), respectively. Cells collected at indicated time points were used for StAR (steroidogenic acute regulatory protein) expressions by Western blotting. Results were normalized to -actin and expressed as ratios relative to the control (NC 16H or NC 8H). *; significant effect by HS (*vs*. NC 16H or NC 8H within the same pharmacological treatment), #; significant effect by time (*vs*. 3H within the same thermal and pharmacological treatment), +; significant effect by Lom+Mip (*vs.* vehicle within the same thermal treatment at the same time), P<0.05, n=3.

**Supplementary Table S1. Heat exposure induces cell death of chicken ovarian granulosa and theca cells.**

| **Item** | **NC** | | | |  | **HS** | | | |  | **Main effect** | | **Interaction** |
| --- | --- | --- | --- | --- | --- | --- | --- | --- | --- | --- | --- | --- | --- |
| **Treatment** | **1H** | **3H** | **8H** | **16H** |  | **1H** | **3H** | **8H** | **16H** | **SEM** | **HS** | **Time** | **HS × Time** |
|  | Granulosa cells | | | | | | | | |  | P-value | | |
| **Cell viability (%)** | 99.4 | 99.0 | 96.7 | 86.5 |  | 99.4^a^ | 97.2^a^ | 33.6^b*^ | 37.4^b*^ | 2.21 | <0.0001 | <0.0001 | <0.0001 |
|  | Theca cells | | | | | | | | |  |  |  |  |
| **Cell viability (%)** | 99.6 | 99.1 | 96.4 | 94.5 |  | 96.8^a^ | 91.8^a^ | 33.6^b*^ | 32.55^b*^ | 1.22 | <0.0001 | <0.0001 | <0.0001 |

| **Item** | **NC** | | |  | **HS3H** | | |  | **Main effect** | | **Interaction** |
| --- | --- | --- | --- | --- | --- | --- | --- | --- | --- | --- | --- |
| **Treatment** | **3H** | **8H** | **16H** |  | **3H** | **3H5R** | **3H13R** | **SEM** | **HS3H** | **Time** | **HS3H × Time** |
|  | Granulosa cells | | | | | | |  | P-value | | |
| **Cell viability (%)** | 96.0 | 94.6 | 91.2 |  | 93.3^a^ | 85.7^a^ | 58.1^b*^ | 3.06 | <0.0001 | <0.0001 | 0.0006 |
|  | Theca cells | | | | | | |  |  |  |  |
| **Cell viability (%)** | 97.7 | 96.4 | 87.7 |  | 86.3 | 85.3 | 80.0 | 3.70 | 0.0038 | 0.0811 | 0.970 |

*; significant effect by HS (vs. NC at the same time point), P<0.05, n=3. Means with different letters (a, b, c) within the same thermal treatment differ significantly among time points, P<0.05, n=3.

**Supplementary Table S2. Effects of heat stress on lipid droplet accumulation of granulosa and theca cells.**

| **Item** | **NC** | | | |  | **HS** | | | |  | **Main effect** | | **Interaction** |
| --- | --- | --- | --- | --- | --- | --- | --- | --- | --- | --- | --- | --- | --- |
| **Treatment** | **1H** | **3H** | **8H** | **16H** |  | **1H** | **3H** | **8H** | **16H** | **SEM** | **HS** | **Time** | **HS × Time** |
|  | Granulosa cells | | | | | | | | |  | P-value | | |
| **Relative intensity**  **(fold, vs. NC1H)** | 1.00^b^ | 1.21^b^ | 2.61^a^ | 2.04^a^ |  | 0.37^a*^ | 0.39^a*^ | 0.18^b*^ | 0.12^b*^ | 0.09 | <0.0001 | <0.0001 | <0.0001 |
|  | Theca cells | | | | | | | | |  |  |  |  |
| **Relative intensity**  **(fold, vs. NC1H)** | 1.00^d^ | 2.04^c^ | 3.02^b^ | 3.97^a^ |  | 2.11^c*^ | 2.97^b*^ | 3.96^a*^ | 1.50^d*^ | 0.11 | 0.6066 | <0.0001 | <0.0001 |

*; significant effect by HS (vs. NC at the same time point), P<0.05, n=3. Means with different letters (a, b, c) within the same thermal treatment differ significantly among time points, P<0.05, n=3.

**Supplementary Table S3. Granulosa and theca cells respond to heat stress for VLDL-apoB secretion.**

| **Item** | **NC** | | |  | **HS3H** | | |  | **Main effect** | | **Interaction** |
| --- | --- | --- | --- | --- | --- | --- | --- | --- | --- | --- | --- |
| **Treatment** | **3H** | **8H** | **16H** |  | **3H** | **3H5R** | **3H13R** | **SEM** | **HS3H** | **Time** | **HS3H × Time** |
|  | Granulosa cells | | | | | | |  | P-value | | |
| **VLDL-apoB secretion**  **(**$\boldsymbol{\mu}$**g/mg protein)** | 0.46^b^ | 0.81^ab^ | 0.92^a^ |  | 2.12^a*^ | 1.89^a*^ | 0.68^b^ | 0.14 | <0.0001 | 0.0152 | 0.0004 |
|  | Theca cells | | | | | | |  |  |  |  |
| **VLDL-apoB secretion**  **(**$\boldsymbol{\mu}$**g/mg protein)** | 0.44^c^ | 0.77^b^ | 1.01^a^ |  | 1.79^b*^ | 2.57^a*^ | 2.11^ab*^ | 0.09 | <0.0001 | 0.0034 | 0.0140 |

*; significant effect by HS (vs. NC at the same time point), P<0.05, n=3. Means with different letters (a, b, c) within the same thermal treatment differ significantly among time points, P<0.05, n=3.

**Supplementary Table S4. Effects of Lomitapide and Mipomersen on VLDL secretion of granulosa and theca cells under heat stress.**

| **Item** | **Vehicle** | |  | **Lom+Mip** | |  |  |  |  |
| --- | --- | --- | --- | --- | --- | --- | --- | --- | --- |
| **Treatment** | **NC16H** | **3H13R** |  | **NC16H** | **3H13R** |  | **Main effect** | | **Interaction** |
|  |  |  |  |  |  | **SEM** | **HS3H** | **Lom+Mip** | **HS3H x Lom+Mip** |
|  | Granulosa cells | | | | |  | P-value | | |
| **VLDL-apoB secretion**  **(**$\boldsymbol{\mu}$**g/mg protein)** | 0.89 | 0.66 |  | 0.18^+^ | 0.13^+^ | 0.05 | 0.0515 | <0.0001 | 0.1891 |
|  | Theca cells | | | | |  |  |  |  |
| **VLDL-apoB secretion**  **(**$\boldsymbol{\mu}$**g/mg protein)** | 1.03 | 1.97^*^ |  | 0.19^+^ | 0.13^+^ | 0.07 | 0.0031 | <0.0001 | 0.0016 |
|  | Granulosa cells | | | | |  | P-value | | |
| **MTTP activity**  **(area under curve, AUC)** | 6881.7 | 3658.2^*^ |  | 4798.4^+^ | 2853.2^*^ | 202.7 | <0.0001 | 0.0003 | 0.0293 |
|  | Theca cells | | | | |  |  |  |  |
| **MTTP activity**  **(area under curve, AUC)** | 5971.2 | 4550.9^*^ |  | 1973.6^+^ | 1327.9^+^ | 287.2 | 0.0080 | <0.0001 | 0.2250 |

*; significant effect by HS (vs. NC at the same time point), P<0.05, n=3. +; significant effect by Lom+Mip (*vs*. vehicle within the same thermal treatment at the same time), P<0.05, n=3.

**Supplementary Table S5. Effects of Lomitapide and Mipomersen on granulosa and theca cell viability under heat stress.**

| **Treatment (NC or HS)** | **Time** | **Lom+Mip** | | **Cell viability**  **(%)** |
| --- | --- | --- | --- | --- |
| Granulosa cells | | | | |
| NC | 3H | Vehicle | | 99.6 |
|  | 16H |  | | 91.3 |
| HS3H | 3H |  | | 91.0 |
|  | 3H13R |  | | 50.3^*#^ |
| NC | 3H | Lom+Mip | | 99.3 |
|  | 16H |  | | 93.9 |
| HS3H | 3H |  | | 88.0 |
|  | 3H13R |  | | 49.9^*#^ |
|  | SEM |  | | 4.69 |
| Source of variation | | | | P-value |
| Main effect | HS3H |  | | <0.0001 |
|  | Time |  | | <0.0001 |
|  | Lom+Mip |  | | 0.9497 |
| Interaction | HS3H x Time | | | 0.0021 |
|  | HS3H x Lom+Mip | | | 0.7447 |
|  | Time x Lom+Mip | |  | 0.7584 |
|  | HS3H x Lom+Mip x Time | | | 0.9876 |
| Theca cells | | | | |
| NC | 3H | Vehicle | | 99.5 |
|  | 16H |  | | 97.0 |
| HS3H | 3H |  | | 88.6 |
|  | 3H13R |  | | 81.1 |
| NC | 3H | Lom+Mip | | 99.2 |
|  | 16H |  | | 89.1 |
| HS3H | 3H |  | | 85.3 |
|  | 3H13R |  | | 79.2 |
|  | SEM |  | | 4.70 |
| Source of variation | | | | P-value |
| Main effect | HS3H |  | | 0.0076 |
|  | Time |  | | 0.1878 |
|  | Lom+Mip |  | | 0.3854 |
| Interaction | HS3H x Time | | | 0.6570 |
|  | HS3H x Lom+Mip | | | 0.9169 |
|  | Time x Lom+Mip |  | | 0.5300 |
|  | HS3H x Lom+Mip x Time | | | 0.3865 |

**Supplementary Table S5. Effects of Lomitapide and Mipomersen on granulosa and theca cell viability under heat stress. (continued)**

| **Treatment (NC or HS)** | **Time** | **Lom+Mip** | | **Cell viability**  **(%)** |
| --- | --- | --- | --- | --- |
| Granulosa cells | | | | |
| NC | 8H | Vehicle | | 99.7 |
|  | 16H |  | | 91.3 |
| HS8H | 8H |  | | 25.7^*^ |
|  | 8H8R |  | | 34.4^*^ |
| NC | 8H | Lom+Mip | | 93.5 |
|  | 16H |  | | 93.9 |
| HS8H | 8H |  | | 86.3^+^ |
|  | 8H8R |  | | 38.9^*#^ |
|  | SEM |  | | 3.89 |
| Source of variation | | | | P-value |
| Main effect | HS8H |  | | <0.0001 |
|  | Time |  | | 0.0022 |
|  | Lom+Mip |  | | 0.0002 |
| Interaction | HS8H x Time | | | 0.0286 |
|  | HS8H x Lom+Mip | | | <0.0001 |
|  | Time x Lom+Mip | |  | 0.0020 |
|  | HS8H x Lom+Mip x Time | | | 0.0001 |
| Theca cells | | | | |
| NC | 8H | Vehicle | | 99.3 |
|  | 16H |  | | 92.3 |
| HS8H | 8H |  | | 40.8^*^ |
|  | 8H8R |  | | 37.1^*^ |
| NC | 8H | Lom+Mip | | 99.4 |
|  | 16H |  | | 99.2 |
| HS8H | 8H |  | | 83.3^*+^ |
|  | 8H8R |  | | 51.7^*#+^ |
|  | SEM |  | | 2.45 |
| Source of variation | | | | P-value |
| Main effect | HS8H |  | | <0.0001 |
|  | Time |  | | 0.0002 |
|  | Lom+Mip |  | | <0.0001 |
| Interaction | HS8H x Time | | | 0.0039 |
|  | HS8H x Lom+Mip | | | <0.0001 |
|  | Time x Lom+Mip | |  | 0.0458 |
|  | HS8H x Lom+Mip x Time | | | 0.0033 |

*; significant effect by HS (vs. NC within the same pharmacological treatment at the same time point), #; significant effect by time (vs. 3H or 8H within the same thermal and pharmacological treatment), +; significant effect by Lom+Mip (vs. vehicle of the same thermal treatment at the same time), P<0.05, n=3.

**Supplementary Table S6. Effects of Lomitapide and Mipomersen on neutral lipid and cholesterol content of granulosa and theca cells under heat stress.**

| **Treatment (NC or HS)** | **Time** | **Lom+Mip** | | **Lipid droplets**  **(fold, vs. NC16H in vehicle)** | | **Total Cholesterol**  **(**$\boldsymbol{\mu}$**g/mg protein)** |
| --- | --- | --- | --- | --- | --- | --- |
| Granulosa cells | | | | | | |
| NC | 3H | Vehicle | | | 1.00 | 9.85 |
|  | 16H |  | | | 1.29^#^ | 11.13 |
| HS3H | 3H |  | | | 0.44^*^ | 2.55^*^ |
|  | 3H13R |  | | | 1.60^*#^ | 22.40^*#^ |
| NC | 3H | Lom+Mip | | | 1.46^+^ | 0.93^+^ |
|  | 16H |  | | | 1.56^+^ | 1.03^+^ |
| HS3H | 3H |  | | | 1.40^+^ | 1.25^+^ |
|  | 3H13R |  | | | 0.90^*#+^ | 1.53^*#+^ |
|  | SEM |  | | | 0.09 | 0.25 |
| Source of variation | | | | | P-value | |
| Main effect | HS3H |  | | | <0.0001 | 0.0007 |
|  | Time |  | | | <0.0001 | <0.0001 |
|  | Lom+Mip |  | | | <0.0001 | <0.0001 |
| Interaction | HS3H x Time | | | | 0.0844 | <0.0001 |
|  | HS3H x Lom+Mip | | | | 0.0049 | 0.0136 |
|  | Time x Lom+Mip | | | | <0.0001 | <0.0001 |
|  | HS3H x Lom+Mip x Time | | | | <0.0001 | <0.0001 |
| Theca cells | | | | | | |
| NC | 3H | Vehicle | | | 1.00 | 2.47 |
|  | 16H |  | | | 1.43^#^ | 1.90^#^ |
| HS3H | 3H |  | | | 1.78^*^ | 3.49^*^ |
|  | 3H13R |  | | | 2.39^*#^ | 3.09^*#^ |
| NC | 3H | Lom+Mip | | | 1.54^+^ | 1.90^+^ |
|  | 16H |  | | | 1.43 | 1.98 |
| HS3H | 3H |  | | | 3.12^*+^ | 1.04^*+^ |
|  | 3H13R |  | | | 0.91^*#+^ | 3.19^*#^ |
|  | SEM |  | | | 0.11 | 0.12 |
| Source of variation | | | | | P-value | |
| Main effect | HS3H |  | | | <0.0001 | <0.0001 |
|  | Time |  | | | 0.0019 | 0.0021 |
|  | Lom+Mip |  | | | 0.2641 | <0.0001 |
| Interaction | HS3H x Time | | | | <0.0001 | <0.0001 |
|  | HS3H x Lom+Mip | | | | 0.0668 | <0.0001 |
|  | Time x Lom+Mip | |  | | <0.0001 | <0.0001 |
|  | HS3H x Lom+Mip x Time | | | | <0.0001 | <0.0001 |

**Supplementary Table S6. Effects of Lomitapide and Mipomersen on neutral lipid and cholesterol content of granulosa and theca cells under heat stress (continued)**

| **Treatment (NC or HS)** | **Lom+Mip** | | | **Lipid droplet**  **(fold, vs. NC16H in vehicle)** | **Total Cholesterol**  **(**$\boldsymbol{\mu}$**g/mg protein)** | |
| --- | --- | --- | --- | --- | --- | --- |
| Granulosa cells | | | | | |  |
| NC 8H | Vehicle | | | 1.00 | 10.49 | |
| HS 8H |  | |  | 0.42^*^ | 0.95^*^ | |
| NC 8H | Lom+Mip | | | 2.09^+^ | 3.00^+^ | |
| HS 8H |  | |  | 0.24^*+^ | 0.67^*^ | |
| SEM | | | | 0.05 | 0.12 | |
| Source of variation | | | | P-value | |  |
| Main effect | | HS8H | | <0.0001 | <0.0001 | |
|  | | Lom+Mip | | 0.0012 | <0.0001 | |
| Interaction | | HS8H x Lom+Mip | | <0.0001 | <0.0001 |  |
| Theca cells | | | | | |  |
| NC 8H | Vehicle | | | 1.00 | 2.64 | |
| HS 8H |  | |  | 3.55^*^ | 1.62^*^ | |
| NC 8H | Lom+Mip | | | 1.30^+^ | 2.52 | |
| HS 8H |  | |  | 0.22^*+^ | 0.45^*+^ | |
| SEM | | | | 0.07 | 0.09 | |
| Source of variation | | | | P-value | |  |
| Main effect | | HS8H | | <0.0001 | <0.0001 | |
|  | | Lom+Mip | | <0.0001 | 0.0005 | |
| Interaction | | HS8H x Lom+Mip | | <0.0001 | 0.0021 |  |

*; significant effect by HS (*vs*. NC within the same pharmacological treatment at the same time point), #; significant effect by time (*vs*. 3H within the same thermal and pharmacological treatment), +; significant effect by Lom+Mip (*vs*. vehicle of the same thermal treatment at the same time), P<0.05, n=3.

**Supplementary Table S7. Effects of Lomitapide and Mipomersen on sex steroid secretion of granulosa and theca cells under heat stress.**

| **Treatment**  **(NC or HS)** | | **Time** | **Lom+Mip** | **Granulosa cells**  **(P4; ng/mg protein)** | **Theca cells**  **(E2; pg/mg protein)** |
| --- | --- | --- | --- | --- | --- |
| NC | | 3H | Vehicle | 4.08 | 64.2 |
|  | | 16H |  | 6.47^#^ | 40.4^#^ |
| HS3H | | 3H |  | 10.47^*^ | 78.7^*^ |
|  | | 3H13R |  | 16.48^*#^ | 135.4^*#^ |
| NC | | 3H | Lom+Mip | 5.18 | 25.8^+^ |
|  | | 16H |  | 7.60^#^ | 66.7^#+^ |
| HS3H | | 3H |  | 10.91^*^ | 145.1^*+^ |
|  | | 3H13R |  | 13.08^*#+^ | 51.1^#+^ |
|  | | SEM |  | 0.48 | 4.92 |
| Source of variation | | | | P-value | |
| Main effect | HS3H | | | <0.0001 | <0.0001 |
|  | Time | | | <0.0001 | 0.3312 |
|  | Lom+Mip | | | 0.6430 | 0.1554 |
| Interaction | HS3H x Time | | | 0.0434 | 0.0159 |
|  | HS3H x Lom+Mip | | | 0.0040 | 0.7816 |
|  | Time x Lom+Mip | | | 0.0253 | 0.0006 |
|  | HS3H x Lom+Mip x Time | | | 0.0229 | <0.0001 |

| **Treatment (NC or HS)** | **Lom+Mip** | | | **Granulosa cells**  **(P4; ng/mg protein)** | **Theca cells**  **(E2; pg/mg protein)** | |
| --- | --- | --- | --- | --- | --- | --- |
| NC 8H | Vehicle | | | 5.27 | 46.2 | |
| HS 8H |  | |  | 19.84^*^ | 72.2^*^ | |
| NC 8H | Lom+Mip | | | 6.22 | 55.0^+^ | |
| HS 8H |  | |  | 14.42^*+^ | 45.2^*+^ | |
| SEM | | | | 0.85 | 2.59 | |
| Source of variation | | | | P-value | |  |
| Main effect | | HS8H | | <0.0001 | 0.0147 | |
|  | | Lom+Mip | | 0.0642 | 0.0081 | |
| Interaction | | HS8H x Lom+Mip | | 0.0157 | 0.0001 |  |

*; significant effect by HS (*vs*. NC within the same pharmacological treatment at the same time), #; significant effect by time (*vs*. 3H within the same thermal and pharmacological treatment), +; significant effect by Lom+Mip (*vs.* vehicle within the same thermal treatment at the same time), P<0.05, n=3.

**Supplementary Table S8. Effects of Lomitapide and Mipomersen on cell proliferation and inflammatory response of granulosa and theca cells under heat stress.**

| **Item** | **Vehicle** | | |  | **Lom+Mip** | | |  |  |  |  |
| --- | --- | --- | --- | --- | --- | --- | --- | --- | --- | --- | --- |
| **Treatment** | **NC16H** | **HS3H** | **3H13R** |  | **NC16H** | **HS3H** | **3H13R** |  | **Main effect** | | **Interaction** |
|  |  |  |  |  |  |  |  | **SEM** | **HS3H** | **Lom+Mip** | **HS3H x Lom+Mip** |
| Granulosa cells | | | | | | | | | P-value | | |
| PCNA expression  (fold, vs. NC16H in vehicle) | 1.00 | 2.09^*^ | 1.68^*#^ |  | 2.02^+^ | 4.41^*+^ | 4.46^*+^ | 0.04 | <0.0001 | <0.0001 | <0.0001 |
| IL-1$\text{β}$ expression  (fold, vs. NC16H in vehicle) | 1.00 | 4.57^*^ | 3.13^*#^ |  | 4.26^+^ | 4.14 | 3.55 | 0.21 | <0.0001 | 0.0002 | <0.0001 |
| Theca cells | | | | | | | | | P-value | | |
| PCNA expression  (fold, vs. NC16H in vehicle) | 1.00 | 1.09 | 0.66^*#^ |  | 1.07 | 1.91^*+^ | 0.10^*#+^ | 0.02 | <0.0001 | 0.0022 | <0.0001 |
| IL-1$\text{β}$ expression  (fold, vs. NC16H in vehicle) | 1.00 | 2.15^*^ | 1.23^#^ |  | 1.15 | 1.10^+^ | 3.39^*#+^ | 0.05 | <0.0001 | <0.0001 | <0.0001 |

**Supplementary Table S8. Effects of Lomitapide and Mipomersen on cell proliferation and inflammatory response of granulosa and theca cells under heat stress. (continued)**

| **Item** | **Vehicle** | |  | **Lom+Mip** | |  |  |  |  |  |
| --- | --- | --- | --- | --- | --- | --- | --- | --- | --- | --- |
| **Treatment** | **NC8H** | **HS8H** |  | **NC8H** | **HS8H** |  | **Main effect** | | **Interaction** |  |
|  |  |  |  |  |  | **SEM** | **HS8H** | **Lom+Mip** | **HS8H x Lom+Mip** |  |
| Granulosa cells | | | | | | | P-value | | | |
| PCNA expression  (fold vs. NC8H in vehicle) | 1.00 | 3.26^*^ |  | 2.01^+^ | 5.21^*+^ | 0.15 | <0.0001 | <0.0001 | 0.0386 | |
| IL-1$\text{β}$ expression  (fold vs. NC8H in vehicle) | 1.00 | 2.11^*^ |  | 4.02^+^ | 5.81^*+^ | 0.11 | <0.0001 | <0.0001 | 0.0323 | |
| Theca cells | | | | | | | P-value | | | |
| PCNA expression  (fold vs. NC8H in vehicle) | 1.00 | 2.75^*^ |  | 1.13 | 2.69^*^ | 0.08 | <0.0001 | 0.7671 | 0.3667 | |
| IL-1$\text{β}$ expression  (fold vs. NC8H in vehicle) | 1.00 | 2.24^*^ |  | 1.14 | 3.32^*+^ | 0.17 | <0.0001 | 0.0104 | 0.0321 | |

*; significant effect by HS (vs. NC 16H or NC 8H within the same pharmacological treatment), #; significant effect by time (vs. 3H within the same thermal and pharmacological treatment), +; significant effect by Lom+Mip (vs. vehicle of the same thermal treatment at the same time), P<0.05, n=3.

**Supplementary Table S9. Effects of Lomitapide and Mipomersen on ROS production and MDA content of granulosa and theca cells under heat stress.**

| **Item** | **Vehicle** | | |  | **Lom+Mip** | | |  |  |  |  |
| --- | --- | --- | --- | --- | --- | --- | --- | --- | --- | --- | --- |
| **Treatment** | **NC16H** | **HS3H** | **3H13R** |  | **NC16H** | **HS3H** | **3H13R** |  | **Main effect** | | **Interaction** |
|  |  |  |  |  |  |  |  | **SEM** | **HS3H** | **Lom+Mip** | **HS3H x Lom+Mip** |
| Granulosa cells | | | | | | | | | P-value | | |
| Mitochondria ROS  (fold, vs. NC16H in vehicle) | 1.00 | 5.05^*^ | 2.35^*#^ |  | 2.33^+^ | 3.30^*+^ | 2.16^#^ | 0.17 | <0.0001 | 0.2263 | <0.0001 |
| Cytosol ROS  (fold, vs. NC16H in vehicle) | 1.00 | 3.68^*^ | 3.86^*^ |  | 1.40 | 4.11^*^ | 3.86^*^ | 0.34 | <0.0001 | 0.4119 | 0.4785 |
| MDA content  (nmol/mg protein) | 0.57 | 2.73^*^ | 1.74^*#^ |  | 1.37^+^ | 2.08^*+^ | 1.57^#^ | 0.10 | <0.0001 | 0.9097 | 0.0002 |
| Theca cells | | | | | | | | | P-value | | |
| Mitochondria RO+S  (fold, vs. NC16H in vehicle) | 1.00 | 2.52^*^ | 4.62^*#^ |  | 5.05^+^ | 4.72^+^ | 4.74 | 0.29 | 0.0014 | <0.0001 | 0.0004 |
| Cytosol ROS  (fold, vs. NC16H in vehicle) | 1.00 | 2.05^*^ | 4.55^*#^ |  | 1.37 | 2.22^*^ | 1.27^#+^ | 0.16 | <0.0001 | 0.0001 | <0.0001 |
| MDA content  (nmol/mg protein) | 0.65 | 2.44^*^ | 3.55^*#^ |  | 1.44^+^ | 1.52^+^ | 2.88^*#+^ | 0.09 | <0.0001 | 0.0206 | <0.0001 |

**Supplementary Table S9. Effects of Lomitapide and Mipomersen on ROS production and MDA content of granulosa and theca cells under heat stress. (continued)**

| **Item** | **Vehicle** | |  | **Lom+Mip** | |  |  |  |  |
| --- | --- | --- | --- | --- | --- | --- | --- | --- | --- |
| **Treatment** | **NC8H** | **HS8H** |  | **NC8H** | **HS8H** |  | **Main effect** | | **Interaction** |
|  |  |  |  |  |  | **SEM** | **HS8H** | **Lom+Mip** | **HS8H x Lom+Mip** |
| Granulosa cells | | | | | | | P-value | | |
| Mitochondria ROS  (fold, vs. NC 8H in vehicle) | 1.00 | 3.02^*^ |  | 1.80^+^ | 3.00^*^ | 0.11 | <0.0001 | 0.0128 | 0.0100 |
| Cytosol ROS  (fold, vs. NC 8H in vehicle) | 1.00 | 3.56^*^ |  | 1.13 | 3.58^*^ | 0.11 | <0.0001 | 0.6008 | 0.7119 |
| MDA content  (nmol/mg protein) | 0.56 | 3.67^*^ |  | 1.50^+^ | 2.36^*+^ | 0.12 | <0.0001 | 0.2048 | <0.0001 |
| Theca cells | | | | | | | P-value | | |
| Mitochondria ROS  (fold, vs. NC 8H in vehicle) | 1.00 | 3.26^*^ |  | 2.07^+^ | 2.27^+^ | 0.12 | <0.0001 | 0.8084 | 0.0001 |
| Cytosol ROS  (fold, vs. NC 8H in vehicle) | 1.00 | 4.98^*^ |  | 1.36 | 3.13^*+^ | 0.27 | <0.0001 | 0.0719 | 0.0151 |
| MDA content  (nmol/mg protein) | 0.85 | 3.01^*^ |  | 1.73^+^ | 2.56^*+^ | 0.08 | <0.0001 | 0.0713 | 0.0002 |

*; significant effect by HS (vs. NC 16H or NC 8H within the same pharmacological treatment), #; significant effect by time (vs. 3H with the same thermal and pharmacological treatment), +; significant effect by Lom+Mip (vs. vehicle within the same thermal treatment at the same time), P<0.05, n=3.

**Supplementary Table S10. Effects of Lomitapide and Mipomersen on StAR expression of granulosa and theca cells under heat stress.**

| **Item** | **Vehicle** | | |  | **Lom+Mip** | | |  |  |  |  |
| --- | --- | --- | --- | --- | --- | --- | --- | --- | --- | --- | --- |
| **Treatment** | **NC16H** | **HS3H** | **3H13R** |  | **NC16H** | **HS3H** | **3H13R** |  | **Main effect** | | **Interaction** |
|  |  |  |  |  |  |  |  | **SEM** | **HS3H** | **Lom+Mip** | **HS3H x Lom+Mip** |
| Granulosa cells | | | | | | | | | | | |
| StAR expression  Fold (vs. NC16H in vehicle) | 1.00 | 1.83^*^ | 1.36^*#^ |  | 0.72^+^ | 2.12^*^ | 0.84^#+^ | 0.05 | <0.0001 | 0.0104 | 0.0002 |
| Theca cells | | | | | | | | | | | |
| StAR expression  Fold (vs. NC16H in vehicle) | 1.00 | 0.85 | 0.43^*#^ |  | 0.39^+^ | 1.79^*+^ | 0.88^*#+^ | 0.07 | <0.0001 | 0.0043 | <0.0001 |

| **Item** | **Vehicle** | |  | **Lom+Mip** | |  |  |  |  |
| --- | --- | --- | --- | --- | --- | --- | --- | --- | --- |
| **Treatment** | **NC8H** | **HS8H** |  | **NC8H** | **HS8H** |  | **Main effect** | | **Interaction** |
|  |  |  |  |  |  | **SEM** | **HS8H** | **Lom+Mip** | **HS8H x Lom+Mip** |
| Granulosa cells | | | | | | | | | |
| StAR expression  Fold (vs. NC8H in vehicle) | 1.00 | 1.61 |  | 0.68 | 2.34 | 0.07 | <0.0001 | 0.1079 | 0.0016 |
| Theca cells | | | | | | | | | |
| StAR expression  Fold (vs. NC8H in vehicle) | 1.00 | 3.02 |  | 0.35 | 1.81 | 0.1  0 | <0.0001 | <0.0001 | 0.0491 |

*; significant effect by HS (*vs*. NC within the same pharmacological treatment at the same time), #; significant effect by time (*vs*. 3H within the same thermal and pharmacological treatment), +; significant effect by Lom+Mip (*vs.* vehicle within the same thermal treatment at the same time), P<0.05, n=3.
